# On “Phantom Tumors” of the Abdomen

**Published:** 1857-07

**Authors:** E. Headlam Greenhow

**Affiliations:** Lecturer on the Public Health at St. Thomas’s Hospital Physician to the Western General Dispensary, etc.


*On "Phantom Tumours" of the Abdomen.* By E. HEADLAM GREENHOW, M. D., Lecturer on the Public Health at St. Thomas's Hospital Physician to the Western General Dispensary, etc.

I desire to bring under the notice of the Society a kind of abdominal tumor, often most embarrassing to the practitioner, and very alarming to the patient, but of which I have been unable to find an account in any publication with which I am acquainted. We are indebted to Dr. Addison for the elucidation of the true nature of these tumors, and, in speaking of them, I shall adopt the name "phantom tumors," which he is accustomed to use in his clinical teaching at Guy's Hospital. During an experience of many years, I only remember to have met with seven or eight cases of the kind, in each of which I was expressly consulted for the tumor, and not for the derangement of health with which it is invariably associated. Probably, as the disordered health on which they depend is of very common occurrence, I should have met with these tumors more frequently had I sought for them. The five cases, the main features of which I intend briefly to detail, had all but one been seen by other practitioners before I was consulted. In the investigation of this excepted case, I had, as will subsequently appear, the benefit of being assisted by a leading metropolitan physician.

The first case of the series came under my notice so long ago as fifteen or sixteen years. The subject of it, a maried lady aged twenty-six, had already borne several children, was in delicate health, and suffered especially from uterine derangement. She was anæmiated, unable to take active exercise, and complained much of anomalous pains, and of tenderness along the course of several large nerves. The greatest source of anxiety, however, was the presence of a tumor in the right lumbar region, apparently about the size of a cricket-ball, but less regularly round. It appeared to be movable, and if attached posteriorly, to be so only by a narrow pedicle. The impression that it conveyed on a manual examination was that of a loose body floating upon or amongst the viscera. In character, the tumor was firm and unyieldiing, free from tenderness, and somewhat changeable in site; for although invariably to be found on examination, its precise relative position varied a little from day to day. I have neglected to note how long the tumor had existed, but several opinions had been taken before I was consulted, and the lady had gone safely and without inconvenience through a pregnancy since its discovery. She had been recommended to place herself in the hands of an eminent surgeon, with a view to the extirpation of the tumor—a procedure to which I most strenuously objected. I have not seen the lady since, but

I know that she has subsequently borne several children, and I learnt several years ago that she was in better health, and had undergone no operation; she is, I believe, alive at the present time. The treatment I adopted—chalybeates, and other means likely to improve the general health—was just what I now believe to have been best suited to the case. It would, however, have been most satisfactory to my patint and her family and very conducive to my own reputation, if I had been able to explain the cause of the tumor, respecting which they were so anxious, and to assure them that it was but a symptom, and an unimportant one, of a troublesome and tedious but not dangerous malady. Although I was unable to form any satisfactory diagnosis of the nature or connections of the tumor in this case, its history served to teach me that there is at least one kind of abdominal tumor that leads to no ill result, and requires no interference--a lesson by no means devoid of practical value.

The next case, which did not present itself until after an interval of several years, was very similar to the preceding one. It also occurred in a married lady, about thirty years of age, who had recovered imperfectly from her last confinement, suffered from profuse leucorrhœa, and was very feeble and unequal to exertion. The tongue was furred, the appetite bad, and the action of the bowels irregular, diarrhœa alternating with constipation. "Has occasional qualmishness and nausea, frequent occipitable headache, and suffers much from abdominal pains unaccompanied by tenderness. She also complains of a contracted sensation across the abdomen." The tumor, which in this case was likewise on the right side, appeared a good deal larger than that already described. Although at first disposed to view it as an ovarian tumor, I abandoned this idea upon a more careful examination, being partly influenced by the circumstance that, although a tumor, apparently as large as a full-sized fœtal head, very plainly existed in the right Iliac region, the abdomen, on careful measurement, was found not to be really larger on that side. Another very remarkable feature in the history of the case, of which I was assured by the patient herself, but the correctness of which I confess to have doubted,—was that the tumor had entirely disappeared previous to and during the period of her last pregnancy, notwithstanding she had been under treament for it at an anterior time. Although in a somewhat different situation, I at once referred this case to the same class as the last, and expressed a hope, based upon that experience, that, however troublesome, the tumor would not prove of any serous consequence. This lady is alive, and in the enjoyment of very tolerable health. She has borne several children since the time of my attendance, but of the tumor I know nothing beyond the fact that it has, as I predicted, led to no unpleasant result.

The third case is that of an unmarried lady, aged between twenty-five and thirty, who was believed to be in a state of hopeless ill-health when she came under my care. The tumor closely resembled both those already described; was more fixed in situation, being in the right hypochondrium; was less movable under examination, and seemed about the size of a large orange. The more prominent symptoms of illness were evidently referable to spinal affection, and under treatment directed to it my patient slowly and gradually recovered. Although I did not at that time understand the connexion between these tumors and spinal disorder, yet relying upon the harmlessness of the tumor in my two previous cases, I treated it as of secondary importance.

The case to which I am now about to refer is, perhaps, the most interesting of the series, for it clearly shows the really unimportant nature of these tumors, and yet how very easily they may be mistaken for examples of serious disease. Mrs. ——, aged forty-four, having borne a family, had suffered for several years from menorrhagia alternating with profuse leucorrhœa. She had also suffered from a variety of other ailments referable to spinal irritation, itself due, I do not doubt, to the disarrangement of the uterine system. I was consulted by her, somewhat more than three years ago, for a tumor in the left hypochondrium, the appearance of which had been long preceded by occasional attacks of pain in that situation, of such intensity as to make her writhe about in bed, and for the relief of which opiates, even in large doses, were of little avail. This pain was of paroxysmal character, often coming on very suddenly, and sometimes without apparent cause, although more frequently as a consequence of over-exertion. It sometimes lasted for many days without intermission, but with variable intensity. The employment of counter-irritation to the spine, and of tonic treatment calculated to improve the general health and lesson the uterine flux, were of essential service; and when, at a subsequent period, I sought for the tumor it was not discoverable. After an interval of many months I was again consulted for the tumor, which sure enough, had very evidently returned, and is described in my notes of the case as "an ovoid movable tumor, free from tenderness, and apparently floating loose in the left hypochondriac region; it is difficult to estimate its size, but it appears to be somewhat reniform, and at least twice the natural size of a kidney." It is further added that the patient was in all other respects in good health; that no fulness, tenderness, or pain existed in the posterior lumbar region, and that the urine was normal. Notwithstanding that I believe the tumor to be of the same character with those already related, I thought it desirable that the patient should have the benefit of a second opinion, particularly as I had been unable to find it on a previous occasion. An eminent physician who was called to my assistance devoted much pains to its elucidation, but without arriving at any more satisfactory conclusion as to its nature than myself. We agreed that it could not be ovarian, from its position; that it was too movable for an enlarged kidney, which was also discountenanced by the absence of any unusual fulness, resistance, or tenderness posteriorly; and that it had not the character, neither had the patient the aspect of malignant disease. Although in great doubt on the subject, we treated it on the supposition that it might eventually prove a hydatid growth. Some time afterwards other symptoms of spinal irritation manifested themselves; and although I had never seen an avowed case of Dr. Addison's "phantom tumors," I began to suspect that this would prove an example of them, as it subsequently did. The patient, very shortly after the consultation went from under my immediate observation, although she continued to act under my instructions. In the course of a few weeks she wrote me word that the tumor had dispersed; and a few months ago, being again in town, she afforded me several opportunities of satisfying myself that the tumor really was gone.

A few weeks since, I was called in to another case of the same description, which has entirely removed any lingering doubt in my mind as to the nature of these tumors. The patient aged thirty-nine, and married for many years without ever being pregnant, has suffered for sixteen or seventeen years from dysmenorrhœa and from several of the various anomolous affections so frequently found in association with uterine functions. She is very prone to attacks of what she calls spasms of the heart; but the ailments which causes most anxiety is a tumor in the left side of the abdomen, just below the margin of the ribs. The tumor is analogous to those already described; is movable, firm, and free from tenderness; but on a careful and somewhat prolonged manipulation, partly frictional, partly kneading, it seems to melt away under the fingers. On examination, very considerable tenderness was found to exist for the space of an inch and a half near the centre of the dorsal vertebræ, pressure by the sides of which produced pain in the chest, and also pain extending round to the left side. Entertaining not the slightest doubt that the tumor here is really a phantom, I have turned my patient's thought from its consideration, assuring her that it is unimportant, and am directing my treatment to the alleviation of the spinal irritation and to the improvement of the general health.

In considering the history of the cases I have described, it is noticeable that all of them were females suffering from some disturbance of the uterine function; and that whilst spinal irration unequivocally existed in three of the patients, its presence may not unfairly be inferred in both the others. Although I have not myself seen any examples of these "phantom tumors" in the male subject, I can easily believe that they may occasionally occur under the influence of slight forms of spinal disease. I half suspect that a medical friend of mine, since dead, who had a tumor in the right hypochondrium, which disappeared for many months, during which he was in the enjoyment of good health, and reapperred at a subsequent time *pari passu* with a return of former bad health, was really the subject of one of these "phantom tumors." That such occurrences are much rarer in men, is readily explicable when we recollect the rarity in them of spinal irritation, of the multifarious symptoms of which these tumors are amongst the most important, since, if not understood, their presence may, as in several of the cases I have related, readily lead to the belief that the patient labors under some very serious disease—ovarian, malignant, or cystoid. The real nature of these tumors is spasmodic; their seat probably the abdominal muscles; for although in every instance I have seen, the tumor appeared to be in the abdominal cavity, the melting away of my last case under manipulation is inconsistent with the belief that they are very deeply seated. Their cause is spinal irritation, the irritated spinal nerves producing spasm in the muscles to which they are distributed. I need scarcely observe how entirely this explanation of their character is in keeping with the history of the tumors in the foregoing cases. If it be admitted that they are formed by the spasmodic contraction of portions of the abdominal muscles, it is no longer matter of surprise that patients suffering from their presence should pass safely through pregnancies; that the tumors should cause no actual enlargement of the abdomen; that they should sometimes disappear spontaneously; that having thus disappeared they should sometimes return; that they should be removed under the use of remedies calculated to improve the general health, and to remedy the cause of the local irritation to which they appeared referable; that they should change their relative position from day to day; or, lastly, that they should be temporarily dispersed under the manipulating hand of the physician. In confirmation of the apparent reality of their presence, and of my assertion as to the embarrassment and anxiety they cause to the practitioner who is ignorant of their true nature, I may point to the fact that the abdomen has been laid open by the surgeon at least five times for the removal of abdominal tumors which were found not to exist. Most prob ably all of these were really examples of these "phantom tumors;" and yet the reality of their existence must, in each of these cases, have been impressed upon the minds of the patients and their relatives, as well as upon that of the operator and his colleagues, before he would have proposed, or they acceded to, so very serious an operation.

I should have been unwilling to bring this subject before the Society in so incomplete a form, and probably would have left it to an abler hand, had I not learnt in conversation with several friends of wide experience, that they had likewise met with examples of these puzzling tumors, without being aware of their true character. I trust that even this imperfect sketch may lead to the clearer elucidation of the subject by directing the attention of other observers towards its investigation, and may serve to avert some of the anxiety and doubt felt by myself when the earlier cases came under my care, as well as of the uneasiness experienecd by persons suffering from a disease apparently of a serious description, but the precise character of which is unknown.—*London Lancet.*

